# Randomized non-inferiority within-patient trial of density gradient processing of sequential ejaculates versus centrifugation-free sperm separation device for ICSI in men with elevated sperm DNA fragmentation- study protocol

**DOI:** 10.3389/fruro.2026.1913330

**Published:** 2026-09-28

**Authors:** Vipin Chandra, Shashank Sanagoudar, Jayesh Shinde, Naval Shah, Isha Suwalka, Sandro C. Esteves

**Affiliations:** 1 Department of Clinical and Lab Operations, Indira IVF Hospital Limited, Udaipur, India; 2 Department of Reproductive Medicine, Indira IVF Hospital Limited, Udaipur, India; 3 Department of Embryology, Indira IVF Hospital Limited, Pune, India; 4 Department of Research and Publication, Indira IVF Hospital Limited, Udaipur, India; 5 ANDROFERT, Andrology and Human Reproduction Clinic, Campinas, Brazil

**Keywords:** centrifugation-free sperm selection, density gradient centrifugation, intracytoplasmic sperm injection, male infertility, non-inferiority trial, sequential ejaculation, sibling-oocyte trial, sperm DNA fragmentation

## Abstract

**Background:**

Pathological sperm DNA fragmentation (SDF) has been associated with impaired fertilization, compromised embryo development, recurrent pregnancy loss, and lower pregnancy rates in assisted reproductive technology (ART). Two same-day strategies proposed to reduce SDF prior to *in vitro* fertilization/intracytoplasmic sperm injection (IVF/ICSI) are: (i) processing a second ejaculate obtained after a short abstinence interval using density gradient centrifugation (DGC), and (ii) selecting sperm from the first ejaculate using a centrifugation-free sperm separation device (CFSS). Although both approaches may enrich sperm populations with lower DNA damage, direct comparative evidence regarding reproductive outcomes remains limited.

**Methods and analysis:**

This prospective, multicenter, within-patient, sibling-oocyte randomized non-inferiority trial will be conducted at nine tertiary ART centers in India. Couples undergoing ICSI will be eligible if the male partner is aged 21–45 years with elevated SDF (DNA fragmentation index >20% by sperm chromatin dispersion assay within 90 days before enrollment) and the female partner is aged 21–35 years and expected to yield at least four metaphase II oocytes. On the day of oocyte retrieval, the first ejaculate collected after 2–3 days of abstinence will undergo processing using the LensHooke^®^ CA0 centrifugation-free sperm separation device, whereas a second ejaculate obtained 1–3 hours later will be processed using conventional DGC. Sibling metaphase II oocytes will be randomized 1:1 to ICSI using sperm from either intervention arm. Embryos will be cultured to the blastocyst stage and graded according to standardized criteria. If both arms generate at least one transferable blastocyst (≥3BB), the embryo selected for the first transfer will be randomized according to its arm of origin; otherwise, transfer will proceed using the available embryo(s) according to a predefined rescue strategy. The primary outcome is ongoing pregnancy rate at 12 weeks’ gestation following transfer of an embryo originating from the randomized study arm. Secondary outcomes include post-processing SDF and sperm parameters, normal fertilization, blastocyst formation and quality, implantation, biochemical and clinical pregnancy. Non-inferiority of DGC processing of the second ejaculate compared with centrifugation-free sperm selection will be assessed using a predefined absolute non-inferiority margin of 10 percentage points.

**Ethics and dissemination:**

The study protocol has been approved by the institutional review boards of all participating centers. Written informed consent will be obtained from all participants. Study findings will be disseminated through peer-reviewed publications and scientific meetings.

**Clinical trial registration:**

https://clinicaltrials.gov/, identifier NCT07626359.

## Introduction

Male infertility contributes to nearly half of all infertility cases worldwide and remains a major challenge in reproductive medicine. Although conventional semen analysis continues to represent the cornerstone of male fertility evaluation, growing evidence indicates that standard semen parameters alone incompletely reflect sperm functional competence and reproductive potential. Among the additional biomarkers proposed to refine male infertility assessment, sperm DNA fragmentation (SDF) has emerged as one of the most extensively investigated indicators of sperm quality and genomic integrity (1, 2).

Oxidative stress is considered the central mechanism underlying sperm DNA damage and may result from several clinical and environmental conditions, including varicocele, infections and inflammation of the male genital tract, aging, environmental and occupational exposures, heat stress, smoking, obesity, and unhealthy lifestyle factors. Additional contributors include defective chromatin remodeling during spermatogenesis and abortive apoptosis. Because mature spermatozoa possess limited DNA repair capacity, damage accumulated during epididymal transit and storage may adversely affect reproductive outcomes despite apparently normal conventional semen parameters (3–5).

Efforts to reduce SDF and improve the selection of genetically intact sperm for ART have led to the development of both medical interventions and advanced sperm selection technologies. The latter have evolved substantially over the past decade. While conventional sperm preparation methods such as density gradient centrifugation (DGC) remain widely used in ART laboratories because they effectively enrich motile spermatozoa and remove cellular debris, leukocytes, and poorly motile sperm (6), centrifugation itself may generate reactive oxygen species and potentially induce additional sperm DNA damage in susceptible samples (7). To overcome these limitations, centrifugation-free sperm separation systems have been developed to mimic more physiologic sperm-selection mechanisms while minimizing mechanical stress during processing (8–11). These platforms enrich for highly motile spermatozoa with improved morphology and lower DNA fragmentation using migration-based selection principles without centrifugation steps (12, 13).

Recent studies and meta-analyses evaluating centrifugation-free or microfluidic sperm-selection systems have demonstrated potential improvements in embryological and reproductive outcomes (10, 12, 14). Nevertheless, available evidence remains heterogeneous, with many studies limited by small sample sizes, non-randomized designs, heterogeneous patient populations not enriched for pathological SDF, and inconsistent outcome reporting. Furthermore, to our knowledge, centrifugation-free sperm selection has not been directly compared against other pragmatic SDF-reduction strategies, such as short abstinence combined with DGC within the same patient population with confirmed pathological levels of SDF.

A direct comparison between centrifugation-free sperm selection applied to the first ejaculate and DGC processing of a second ejaculate obtained after short abstinence is clinically relevant for several reasons. Both interventions are relatively accessible, can be performed on the day of oocyte retrieval, and aim to improve sperm genomic integrity before ICSI without resorting to invasive sperm retrieval procedures. However, the relative effectiveness of these approaches in improving laboratory and clinical reproductive outcomes remains uncertain (6, 15).

The present study was therefore designed as a prospective multicenter within-patient sibling-oocyte randomized non-inferiority trial to compare two same-day sperm preparation strategies in couples undergoing ICSI in whom the male partner exhibits elevated SDF. Specifically, the study aims to determine whether DGC processing of a second ejaculate obtained after very short abstinence is non-inferior to centrifugation-free sperm selection applied to the first ejaculate with respect to ongoing pregnancy rates, while also evaluating their effects on post-processing sperm DNA integrity, fertilization, blastocyst development, implantation, and pregnancy outcomes.

## Methods

### Study design and setting

This study is designed as a prospective, multicenter, within-patient, sibling-oocyte randomized non-inferiority trial conducted across nine tertiary ART centers in India. The study will compare two same-day sperm preparation strategies for ICSI in couples in whom the male partner has elevated sperm DNA fragmentation.

The trial uses a within-patient design in which sibling metaphase II (MII) oocytes retrieved during the same ovarian stimulation cycle are randomly allocated to ICSI using sperm prepared by either: (i) centrifugation-free sperm separation of the first ejaculate (Arm A), or (ii) DGC processing of a second ejaculate collected after a very short abstinence interval (Arm B). This design minimizes confounding arising from female-related factors, ovarian response variability, and inter-cycle laboratory differences.

The study is designed as a non-inferiority trial because density gradient centrifugation of a second ejaculate represents a simple, low-cost, and widely accessible intervention that may provide reproductive outcomes comparable to centrifugation-free microfluidic sperm preparation.

### Participants

Couples undergoing ICSI treatment will be screened for eligibility at participating centers.

#### Inclusion criteria

Eligible couples must meet all male and female partner criteria described below.

Male partners must be aged 21 to 45 years and have elevated SDF, defined as an SDF rate >20%, measured by sperm chromatin dispersion assay within 90 days before enrollment. They must also be able to provide two ejaculates on the day of oocyte retrieval, and the ejaculated sperm sample must be suitable for ICSI.

Female partners must be aged 21 to 35 years, planned to undergo an autologous ICSI cycle, and expected to retrieve at least four metaphase II oocytes. In addition, they must have a normal uterine cavity assessment according to local clinical practice.

#### Exclusion criteria

Couples will be excluded if any of the following criteria are present: use of surgically retrieved spermatozoa; severe oligozoospermia (<5 million/mL), severe asthenozoospermia (<20% progressive motility), or azoospermia precluding allocation of sperm to both intervention arms; use of donor oocytes; planned preimplantation genetic testing; untreated hydrosalpinx; severe uterine abnormalities expected to impair implantation; uncontrolled endocrine or systemic disease in either partner; or inability or unwillingness to provide informed consent.

### Screening sperm DNA fragmentation testing for eligibility confirmation

Sperm DNA fragmentation (SDF) testing will be performed before enrollment to confirm eligibility, using a harmonized sperm chromatin dispersion assay protocol across all participating centers with the PrimeSperm^®^ SCD kit (Prime Healthcare, India).

Before study initiation, all laboratories will undergo pre-study calibration and standardization procedures to ensure consistency in assay performance and interpretation. Internal quality-control measures will include weekly assessment of high- and low-DFI reference samples, duplicate scoring of at least 10% of slides, and acceptable inter-observer variation defined as ≤10%. In addition, pre-study inter-laboratory agreement will be assessed, with a target intraclass correlation coefficient or kappa value of ≥0.70.

DNA fragmentation index (DFI) will be calculated as the percentage of spermatozoa exhibiting fragmented DNA among at least 400 scored spermatozoa. SDF testing will be repeated if protocol deviations occur, including incorrect abstinence duration, processing delays exceeding 60 minutes, or inappropriate sample temperature conditions.

### Day-of-procedure semen collection and sample allocation

On the day of oocyte pick-up, the male partner will provide the first semen sample after 2–3 days of sexual abstinence. After complete liquefaction, an initial semen analysis will be performed to confirm the availability of sufficient sperm concentration and motility for ICSI and for allocation to the CA0 arm.

The first ejaculate will be processed using the LensHooke^®^ CA0 sperm separation device. A second ejaculate will then be collected 1–3 hours after the first semen sample and processed using density gradient centrifugation. Semen analysis and SDF testing will be performed before and after sperm preparation in both arms to evaluate the effects of each preparation strategy on semen parameters and SDF.

#### Arm 1: CA0 sperm preparation

Sperm preparation in Arm 1 will be performed using the LensHooke^®^ CA0 microfluidic sperm separation device (Bonraybio Co., Ltd., Taiwan), according to the manufacturer’s instructions. Briefly, 1.0 mL of liquefied semen will be loaded into the lower chamber of the device, followed by 0.9 mL of pre-equilibrated flushing medium (ORIGIO/SAGE; CooperSurgical, USA), which will be added to the upper chamber using a pipette.

The device will then be incubated at 37 °C for a minimum of 20 minutes to allow motile sperm to migrate into the upper chamber through a 10-µm polycarbonate membrane. After incubation, 0.5 mL of the recovered sperm suspension will be carefully aspirated from the upper chamber and subsequently used for ICSI.

#### Arm 2: density gradient centrifugation of the second ejaculate

A second ejaculate will be collected 1–3 hours after the first semen sample. After complete liquefaction, sperm preparation will be performed using a two-layer density gradient centrifugation protocol comprising 40% and 80% silane-coated silica gradients suspended in balanced salt medium (SAGE/ORIGIO; CooperSurgical, USA).

Briefly, 1–2 mL of each gradient solution will be carefully layered into a sterile 15-mL conical tube (SPL Life Sciences Co., Ltd., South Korea), with the 80% fraction placed at the bottom and the 40% fraction layered above it. Subsequently, 1–2 mL of liquefied semen will be gently overlaid onto the gradient column and centrifuged at 400*g* for 15 minutes at room temperature.

After centrifugation, the supernatant will be discarded, and the sperm pellet will be transferred to a sterile 5-mL round-bottom tube (SPL Life Sciences Co., Ltd., South Korea) containing 2 mL of flushing medium (ORIGIO/SAGE; CooperSurgical, USA). The pellet will then be gently resuspended and centrifuged at 500*g* for 5 minutes at room temperature. After this washing step, the supernatant will be carefully removed, and the final sperm pellet will be resuspended in 0.5 mL of flushing medium. The prepared sperm suspension will subsequently be used for ICSI.

All participating centers will follow harmonized operating procedures for abstinence verification, semen liquefaction, initial semen analysis, sample allocation, incubation conditions, media preparation, sample handling, centrifugation settings, and timing of sperm preparation.

### Assessment of semen parameters and sperm DNA fragmentation before and after sperm preparation

For both study arms, semen analysis and SDF testing will be performed before and after sperm preparation to evaluate the effects of each sperm preparation method on semen parameters and SDF. Sperm DNA fragmentation will be assessed using the Prime Sperm^®^ DNA Fragmentation Test Kit (Prime Healthcare, India), which is based on the sperm chromatin dispersion (SCD) principle. Trained laboratory personnel blinded to study outcomes will perform slide evaluation according to predefined quality-control procedures. DNA fragmentation index will be calculated as the percentage of spermatozoa exhibiting fragmented DNA among at least 400 scored spermatozoa.

In Arm 1, assessments will be performed on the first ejaculate before CA0 processing and on the recovered motile sperm fraction after CA0 processing. In Arm 2, assessments will be performed on the second ejaculate before DGC and repeated after completion of sperm preparation.

Outcomes from these paired pre- and post-processing assessments will be used to compare the ability of each sperm preparation strategy to improve motility, sperm concentration or recovery, and SDF levels before ICSI. All participating centers will follow harmonized procedures for semen handling, timing of assessments, sperm preparation, and post-processing sample recovery.

### Ovarian stimulation and oocyte handling

Controlled ovarian stimulation, ovulation triggering, and oocyte pick-up will be performed according to standard clinical practice at each participating center. Oocyte retrieval will be carried out 34–36 hours after ovulation trigger.

Following retrieval, cumulus–oocyte complexes will be incubated under standard laboratory conditions and subsequently denuded according to local procedures. Only mature metaphase II oocytes will be included in the sibling-oocyte allocation and used for ICSI.

### Randomization and allocation

After confirmation of the minimum eligibility number of metaphase II oocytes, sibling oocytes from each participant will be randomly allocated in a 1:1 ratio to insemination with sperm prepared by either Arm 1 or Arm 2.

Randomization will be performed at the oocyte level to ensure within-patient comparison and to minimize the influence of female-related prognostic factors. When an odd number of mature oocytes is available, the additional oocyte will be allocated according to the pre-specified randomization sequence.

### Intracytoplasmic sperm injection and embryo culture

ICSI will be performed using standard micromanipulation procedures at each participating center. For each randomized oocyte, spermatozoa will be selected from the corresponding allocated preparation arm. Injected oocytes will be cultured under standard laboratory conditions, and fertilization will be assessed 16–18 hours after ICSI. Normal fertilization will be defined by the presence of two pronuclei and two polar bodies.

Embryo culture will continue according to local laboratory protocols to the blastocyst stage. Embryo development and quality will be assessed using standardized morphological criteria at each participating center.

### Embryo transfer policy

Embryo transfer will follow a predefined randomized strategy. If both study arms generate at least one transferable blastocyst of grade ≥3BB, the embryo selected for the first transfer will be randomly chosen between the two arms using a computer-generated allocation sequence. Embryo selection within each arm will follow local clinical practice, prioritizing the embryo with the highest morphological grade.

If only one study arm generates at least one transferable blastocyst (≥3BB), the first embryo transfer will be performed using the available embryo(s) from that arm, and the opposite arm will be recorded as having no transferable embryo available for transfer.

All supernumerary blastocysts will be vitrified according to local laboratory protocols and may be used in subsequent frozen embryo transfer cycles.

### Rescue strategy

#### Sperm selection

If one sperm preparation arm yields an insufficient number of motile spermatozoa to permit ICSI for the allocated oocytes, the embryology team may use spermatozoa from the other preparation arm as a rescue strategy to avoid compromising patient care.

Any rescue use of sperm across arms will be documented, including the reason for rescue, the number of oocytes affected, and the final sperm source used for injection. These oocytes or embryos will be analyzed according to the pre-specified statistical plan, with appropriate consideration in per-protocol and sensitivity analyses.

#### Embryo transfer

Given the sibling-oocyte design, situations may arise in which one intervention arm fails to generate a transferable blastocyst while the other arm produces one or more embryos suitable for transfer. In such cases, embryo transfer will proceed using the available embryo(s) from the arm generating transferable blastocysts to avoid compromising patient care.

These cycles will remain in the modified intention-to-treat analysis population. For the primary outcome analysis, ongoing pregnancy will be attributed to the arm from which the transferred embryo originated. The opposite arm will be classified as having failed to produce a transferable embryo. This approach preserves the pragmatic nature of the study while ensuring that patients are not denied embryo transfer opportunities because of study allocation.

The study flowchart is summarized in **Figure 1**.

**Figure 1:**
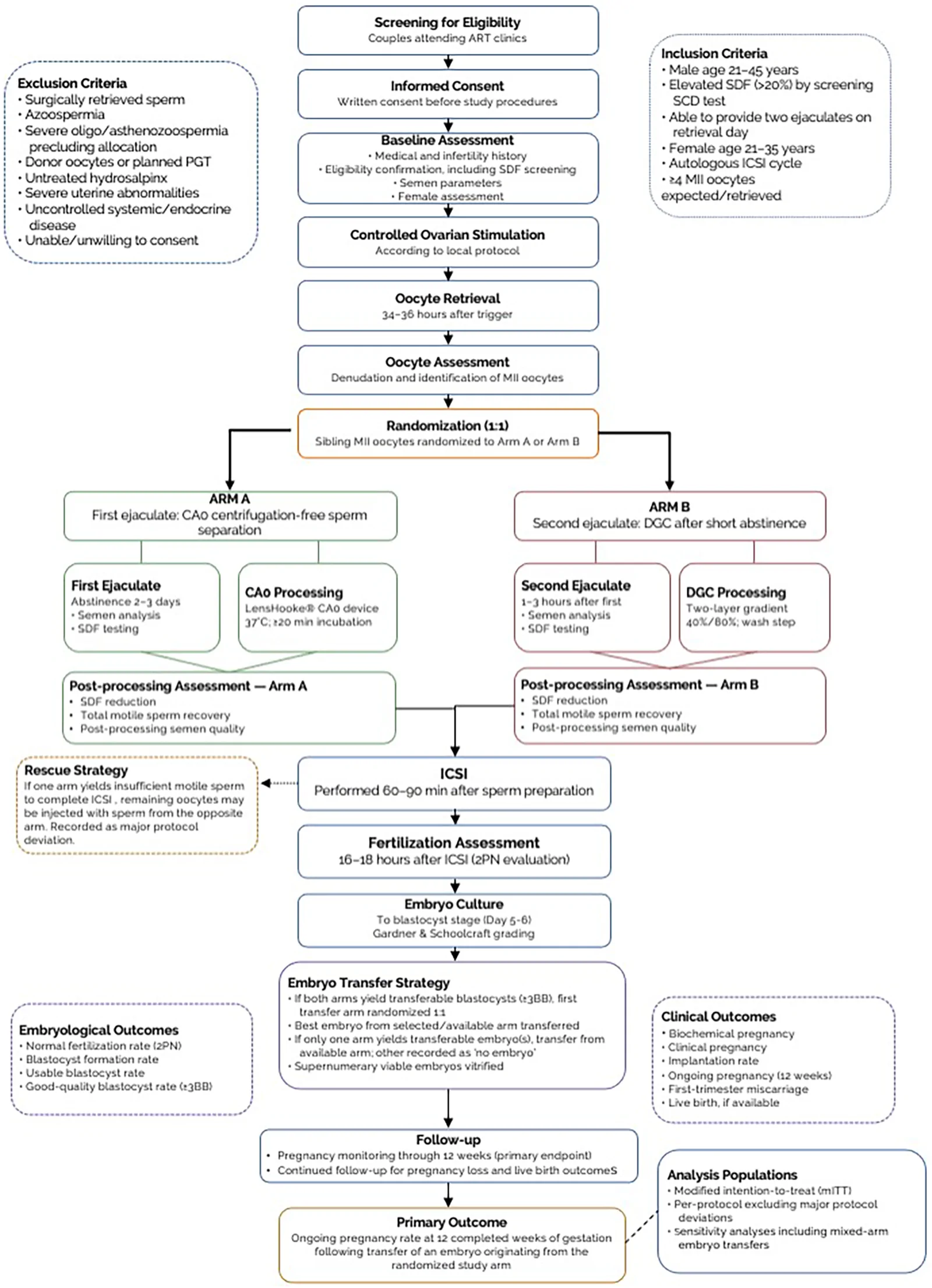
Study flow diagram of the randomized within-patient non-inferiority trial comparing CA0 centrifugation-free sperm separation and density gradient centrifugation of a second ejaculate in couples undergoing ICSI in men with elevated sperm DNA.

### Outcomes

The primary outcome will be ongoing pregnancy rate, defined as the presence of at least one viable intrauterine pregnancy with fetal cardiac activity at 12 completed weeks of gestation following transfer of an embryo originating from the randomized study arm.

Secondary outcomes will include laboratory, embryological, clinical, and safety outcomes (**Table 1**). Laboratory outcomes will assess the effect of each sperm preparation strategy on sperm concentration, motility, total motile sperm recovery, DFI, and relative SDF reduction following sperm preparation. Embryological outcomes will evaluate normal fertilization, blastocyst formation, usable blastocyst yield, and blastocyst quality. Clinical outcomes will include implantation, biochemical pregnancy, clinical pregnancy, pregnancy loss, and live birth rates. Safety and feasibility outcomes will include cycle cancellation, absence of usable sperm after preparation, rescue sperm use across study arms, and adverse reproductive outcomes, when available.

**Table 1:** Study outcomes and definition.

Outcome category	Outcome	Definition / assessment	Unit of analysis or denominator
**Primary outcome**	Ongoing pregnancy rate	Presence of at least one viable intrauterine pregnancy with fetal heartbeat beyond 10–12 weeks of gestation	Per transfer of an embryo originating from the randomized study arm
**Semen laboratory outcomes**	Sperm concentration	Sperm concentration measured before and after sperm preparation	Pre- and post-processing semen samples
	Total and progressive motility	Total and progressive sperm motility measured before and after sperm preparation	Pre- and post-processing semen samples
	Total motile sperm count / recovery	Total number of motile spermatozoa recovered after preparation	Post-processing sperm fraction
	Sperm DNA fragmentation	DNA fragmentation index (DFI) measured before and after sperm processing using the Prime Sperm® DNA Fragmentation Test Kit based on the sperm chromatin dispersion method	Pre- and post-processing semen samples
	Relative SDF reduction	Percentage reduction in SDF after sperm preparation compared with the corresponding pre-processing sample	Paired pre-/post-processing samples
**Embryological outcomes**	Normal fertilization rate	Proportion of injected MII oocytes with two pronuclei and two polar bodies at 16–18 hours after ICSI	Per injected MII oocyte
	Cleavage rate	Proportion of normally fertilized oocytes reaching cleavage-stage development	Per normally fertilized oocyte, assessed at day 3 after ICSI
	Blastocyst formation rate	Proportion of normally fertilized oocytes developing into blastocysts	Per normally fertilized oocyte, assessed days 5-6 after ICSI
	Usable blastocyst rate	Proportion of blastocysts suitable for transfer or cryopreservation according to local laboratory criteria	Per injected MII oocyte and/or per normally fertilized oocyte, assessed days 5-6 after ICSI
	Good-quality blastocyst rate	Proportion of blastocysts graded ≥3BB according to the Gardner and Schoolcraft classification system	Per embryo
**Clinical outcomes**	Biochemical pregnancy rate	Positive serum β-hCG after embryo transfer	Per embryo transfer
	Clinical pregnancy rate	Presence of an intrauterine gestational sac on ultrasound	Per embryo transfer
	Implantation rate	Number of gestational sacs observed divided by the number of embryos transferred	Per embryo transferred
	Pregnancy loss rate	Loss of a biochemical or clinical pregnancy before viability, according to local definitions	Per pregnancy
	Live birth rate	Delivery of at least one live-born infant	Per embryo transfer
**Safety and feasibility outcomes**	Cycle cancellation	Cancellation before embryo transfer due to laboratory, clinical, or patient-related reasons	Per initiated cycle
	Absence of usable sperm after preparation	Failure of a sperm preparation arm to yield sufficient motile spermatozoa for ICSI	Per preparation arm
	Rescue sperm use	Use of spermatozoa from the alternate preparation arm to avoid compromising ICSI	Per oocyte and per cycle
	Adverse reproductive outcomes	Ectopic pregnancy, severe ovarian hyperstimulation syndrome, or other clinically relevant adverse events, when available	Per cycle or per pregnancy

### Sample size calculation

The sample size calculation was based on the primary endpoint of OPR following transfer of an embryo originating from either the centrifugation-free sperm separation arm or the density gradient centrifugation arm according to the predefined embryo-transfer randomization strategy.

The study is designed as a non-inferiority trial comparing ongoing pregnancy rates between the two sperm preparation strategies. The non-inferiority margin (Δ) was defined as an absolute difference of 10 percentage points in ongoing pregnancy rate between the two intervention arms. Density gradient centrifugation processing of the second ejaculate will be considered non-inferior if the ongoing pregnancy rate is not more than 10 percentage points lower than that achieved using centrifugation-free sperm separation of the first ejaculate.

Sample size estimation was based on the formula for comparison of two proportions in a non-inferiority trial:


$$n={\left(Z_{1-\alpha }+Z_{1-\beta }\right)}^{2}\times [p1(1-p1)+p2(1-p2)]/\Delta 2$$

where:

- n = required sample size per group;
- Z_1−α_ = standard normal deviate corresponding to the one-sided significance level;
- Z_1−β_ = standard normal deviate corresponding to study power;
- p1 and p2 = expected ongoing pregnancy rates in the two groups;
- Δ = predefined non-inferiority margin.

Because reliable estimates of within-couple correlation for the primary endpoint are unavailable, sample size calculations were conservatively based on standard formulas for comparison of two independent proportions. This approach is expected to provide a conservative estimate of the required number of embryo transfers.

The following assumptions were adopted:

- Expected ongoing pregnancy rate per transfer of 40% in both groups;
- One-sided alpha level of 0.025;
- Statistical power of 80%;
- Equal allocation ratio (1:1);
- Non-inferiority margin of 10 percentage points.

Substituting these assumptions into the formula:


$$n={\left(1.96\;+\;0.84\right)}^{2}\times [(0.40)\left(0.60\right)+\left(0.40\right)\left(0.60\right)]/{\left(0.10\right)}^{2}$$

yielded an estimated sample size of approximately 377 embryo transfers per arm. To compensate for potential attrition, incomplete embryo-transfer data, and protocol deviations, the sample size was increased by approximately 10%, resulting in a final target enrollment of approximately 415 embryo transfers attributable to each study arm (830 total). The number of enrolled cycles may need to be higher depending on cancellations, no-transfer cycles, and mixed-arm transfers.

Non-inferiority will be concluded if the lower bound of the two-sided 95% confidence interval for the absolute difference in ongoing pregnancy rates between groups remains above −10% in both modified intention-to-treat and per-protocol analyses.

### Statistical analysis

For the primary arm-level analysis, OPR will be attributed to the sperm preparation arm from which the transferred embryo originated. Therefore, the primary analysis population will include embryo transfers with clear arm attribution, defined as either single embryo transfer or transfer of multiple embryos derived from the same sperm preparation arm.

Cycles in which embryos derived from both study arms are transferred together will not be included in the primary arm-attributed OPR comparison, because the pregnancy outcome cannot be unequivocally assigned to either intervention arm. These mixed-arm transfers will be analyzed separately in pre-specified sensitivity or descriptive analyses.

The primary comparison of OPR between CA0 and DGC will be performed using a generalized linear model with binomial distribution and logit link, or an equivalent logistic regression method. The absolute risk difference (DGC minus CA0) and its corresponding two-sided 95% confidence interval will be estimated. Non-inferiority will be concluded if the lower limit of the confidence interval remains above −10%.

If more than one embryo transfer from the same randomized treatment cycle contributes to the analysis, such as subsequent frozen embryo transfers involving embryos from the same study arm, statistical models will account for within-patient clustering using generalized estimating equations (GEE) or mixed-effects logistic regression models, as appropriate.

Secondary laboratory and embryological outcomes will be analyzed according to the original oocyte allocation arm. These outcomes will include semen parameters, sperm DNA fragmentation before and after sperm preparation, relative SDF reduction, fertilization rate, cleavage rate, blastocyst formation rate, usable blastocyst rate, embryo quality, and cryopreservation rate. Because sibling oocytes from the same participant are allocated to both intervention arms, analyses of oocyte- and embryo-level outcomes will account for within-patient correlation using paired statistical methods, generalized estimating equations, or mixed-effects regression models, as appropriate. Participant identification will be included as a random effect whenever multiple oocytes or embryos from the same individual contribute to the analysis.

Continuous variables will be summarized as mean ± standard deviation or median and interquartile range, depending on distribution. Categorical variables will be summarized as frequencies and percentages. Between-arm comparisons of paired continuous outcomes will be performed using paired t-tests or Wilcoxon signed-rank tests, as appropriate. Categorical paired outcomes will be compared using McNemar’s test, generalized estimating equations, or mixed-effects logistic regression, depending on the structure of the data.

The primary efficacy analysis will follow a modified intention-to-treat (mITT) principle. The mITT population will include all randomized treatment cycles resulting in at least one embryo transfer with attributable arm origin, irrespective of protocol deviations occurring after oocyte allocation. This approach preserves the randomized nature of the study while reflecting the clinical realities of a laboratory-based sibling-oocyte design.

Supportive per-protocol analyses will be performed after exclusion of major protocol deviations, including incorrect oocyte allocation, rescue sperm use across study arms, failure to perform the assigned sperm preparation procedure, incorrect embryo-transfer allocation, or embryo transfers without clear arm attribution. Major protocol deviations defining the per-protocol population will be specified before database lock in a predefined statistical analysis plan.

The analysis is considered modified intention-to-treat rather than strict intention-to-treat because only randomized cycles in which at least one metaphase II oocyte undergoes ICSI according to a study intervention can contribute meaningfully to the primary efficacy analysis. This approach is appropriate given the laboratory-based nature of the sibling-oocyte design and the possibility of unavoidable exclusions before meaningful exposure to the allocated intervention, including absence of mature oocytes despite planned enrollment, inability to obtain the second ejaculate, technical laboratory failure before ICSI, cancellation before oocyte allocation, or absence of embryo transfer.

Participating center will be included as a fixed effect in multivariable models whenever appropriate to account for potential inter-center variability. Missing primary outcome data are expected to be uncommon because pregnancy outcomes are routinely captured during clinical follow-up. No formal imputation will be performed for the primary analysis; however, sensitivity analyses may be conducted if missing outcome data exceed 5% of the study population.

All statistical tests will be two-sided with a significance level of 0.05, except for the primary non-inferiority analysis, which will follow the pre-specified one-sided non-inferiority scheme. Statistical analyses will be performed using JMP^®^ Pro, (SAS Institute Inc., Cary, NC, USA), and/or IBM SPSS Statistics (IBM Corp., Armonk, NY, USA).

## Discussion

### Generalizability

The multicenter design of this trial, involving tertiary ART centers in India, is expected to enhance the external validity and generalizability of the findings. By including centers with established IVF and andrology laboratory infrastructure, the study will reflect real-world clinical practice while maintaining harmonized procedures for semen analysis, SDF testing, sperm preparation, oocyte allocation, ICSI, and embryo culture.

The study population includes couples undergoing autologous ICSI in whom the male partner has elevated SDF, a clinically relevant subgroup frequently encountered in ART practice. The inclusion of women aged 21–35 years and cycles with an expected minimum number of mature oocytes is intended to reduce confounding from female-related prognostic factors and ensure feasibility of sibling-oocyte allocation. However, this eligibility scheme may limit extrapolation to older women, poor ovarian responders, donor-oocyte cycles, surgically retrieved sperm cases, severe oligozoospermia or azoospermia, and cycles undergoing preimplantation genetic testing.

The comparison between CA0 centrifugation-free sperm separation and DGC processing of a second ejaculate is particularly relevant to resource-variable ART settings. If DGC of a second ejaculate demonstrates non-inferior reproductive outcomes, the findings may support a pragmatic, low-cost alternative for laboratories that do not routinely have access to centrifugation-free sperm selection technologies. Conversely, if CA0 demonstrates superior outcomes, the results would provide clinically meaningful evidence to guide the adoption of centrifugation-free sperm preparation in selected patients with elevated SDF.

### Expected impact of the proposed trial

This trial addresses an important and clinically relevant question in reproductive medicine: whether the use of a second ejaculate processed by conventional DGC can achieve ongoing pregnancy outcomes comparable to those obtained with the CA0 centrifugation-free sperm preparation in couples with elevated SDF undergoing ICSI.

The study has the potential to inform clinical decision-making by clarifying whether a simple modification in semen collection strategy, i.e., obtaining a second ejaculate after a short abstinence interval, can provide a feasible alternative to specialized sperm separation devices. This is especially relevant in settings where access to advanced sperm preparation technologies may be limited by cost, availability, or laboratory workflow constraints.

In addition to the primary clinical endpoint, the paired pre- and post-processing assessments of semen parameters and SDF will provide mechanistic insight into how each strategy affects sperm quality before ICSI. The sibling-oocyte design will further allow direct comparison of fertilization, embryo development, blastocyst formation, and embryo usability while minimizing confounding from female-related factors.

If the second-ejaculate DGC strategy is shown to be non-inferior to CA0, the findings could support a widely accessible, low-cost, and easily implementable approach for managing elevated SDF in ICSI cycles. If non-inferiority is not demonstrated, the results will still be valuable by defining the limits of second-ejaculate DGC and strengthening the evidence base for centrifugation-free sperm selection in this patient population.

Overall, the proposed trial is expected to contribute high-quality prospective evidence to guide laboratory and clinical management of couples undergoing ICSI in the context of elevated SDF.

### Dissemination plan

The findings of this study will be disseminated through peer-reviewed scientific publication and presentation at national and international conferences in reproductive medicine, andrology, embryology, and assisted reproduction. Results will be reported in accordance with applicable reporting guidelines for randomized and non-inferiority trials.

Study outcomes will be communicated regardless of whether the primary hypothesis is confirmed. Both positive and negative findings will be considered clinically relevant, given the practical implications of comparing a low-cost second-ejaculate strategy with centrifugation-free sperm preparation.

Authorship and publication decisions will follow standard international authorship criteria. Participating centers will have the opportunity to contribute to data interpretation, manuscript preparation, and dissemination activities according to their role in the study. Where appropriate, study findings may also be incorporated into educational activities, laboratory protocols, and future clinical practice recommendations for the management of elevated SDF in ART.

### Limitations

This trial has several limitations that should be acknowledged. First, although the sibling-oocyte design minimizes confounding arising from female-related factors, ovarian stimulation characteristics, and inter-cycle variability for laboratory and embryological outcomes, complete clinical pairing may not always be achievable. Embryo transfer randomization is performed only when both study arms generate at least one transferable blastocyst (≥3BB). When transferable embryos are available from only one arm, a predefined rescue transfer strategy will be implemented to avoid compromising patient care. Consequently, some clinical outcomes will derive from transfers originating from a single study arm, which may reduce the degree of within-patient comparability for the primary endpoint.

Second, the study population is restricted to couples undergoing autologous ICSI in whom the female partner is aged 21–35 years and expected to yield at least four mature oocytes. Although this design improves internal validity and facilitates sibling-oocyte allocation, it may limit generalizability to older women, poor ovarian responders, donor-oocyte cycles, cycles using surgically retrieved spermatozoa, men with severe oligozoospermia or azoospermia, and cycles undergoing preimplantation genetic testing.

Third, SDF will be assessed using a harmonized SCD assay protocol across participating centers. Despite extensive standardization, calibration, and quality-control procedures, some degree of inter-laboratory variability may remain unavoidable. Similarly, ovarian stimulation protocols, embryo culture conditions, and embryo transfer practices will follow local clinical standards, potentially introducing center-level variability. This limitation will be mitigated through protocol harmonization and adjustment for participating center in statistical analyses when appropriate.

Fourth, the interventions differ not only in sperm preparation method but also in ejaculate source. The centrifugation-free sperm separation strategy is applied to the first ejaculate obtained after 2–3 days of abstinence, whereas density gradient centrifugation is performed on a second ejaculate collected after a short abstinence interval. Therefore, any observed differences may reflect the combined effects of ejaculate sequence, abstinence duration, and sperm preparation technique rather than the isolated effect of sperm processing alone. Nevertheless, this design directly reflects the pragmatic clinical question addressed by the trial and mirrors how these interventions would be implemented in routine clinical practice.

Finally, although ongoing pregnancy rate was selected as the primary endpoint because of its strong clinical relevance and feasibility, live birth remains the ultimate measure of reproductive success. The study is not specifically powered to detect differences in live birth rate; therefore, analyses of live birth outcomes should be interpreted as exploratory and hypothesis-generating.
